# Early inflammatory and nutritional marker recovery patterns and short-term outcomes in critically ill older adults: a 72-h landmark cohort study

**DOI:** 10.3389/fmed.2026.1919790

**Published:** 2026-09-04

**Authors:** Yue Zheng, Kang Han, Dan Wang

**Affiliations:** 1 Geriatric Intensive Care Unit, The Fourth Affiliated Hospital of Nanjing Medical University, Nanjing, China; 2 Intensive Care Unit, The Fourth Affiliated Hospital of Nanjing Medical University, Nanjing, China

**Keywords:** critical illness, frailty, inflammatory markers, landmark analysis, mortality, nutritional markers, older adults, trajectory

## Abstract

**Background:**

Admission severity scores describe acute organ dysfunction but may not capture early recovery in older intensive care unit (ICU) patients. We examined whether investigator-defined changes in routinely measured inflammatory and nutritional markers during the first 72 h identified distinct recovery patterns associated with short-term outcomes.

**Methods:**

This single-center retrospective cohort included patients aged 65 years or older admitted from January 5, 2021, through May 31, 2026, who survived to a 72-h landmark and had first-window measurements at ICU admission (T0, 0–24 h) and 48–72 h (T1). Four rule-based recovery patterns were defined and assigned using internally derived marker thresholds and fixed directional/count rules finalized without reference to outcomes. The primary outcome was mortality from the landmark to day 28. Cox models sequentially adjusted for demographic factors, baseline clinical severity, and geriatric vulnerability. Within each bootstrap sample, pooled marker thresholds were re-estimated, patients were reassigned using the fixed operational rules, and the outcome models were refitted.

**Results:**

Among 934 screened admissions, 620 patients entered the landmark cohort and 122 died by day 28. Pattern 3 (initial extreme burden without improvement-rule fulfillment) included 180 patients and had the highest crude mortality (31.7%, 57/180), versus 9.8% (25/255) in Pattern 1. In the geriatric-adjusted model, the overall four-level association with mortality was not significant (likelihood-ratio *p* = 0.196), and none of the three non-reference patterns was independently associated with mortality; the hazard ratio for Pattern 3 versus Pattern 1 was 1.64 (95% confidence interval, 0.97–2.79; *p* = 0.066). In exploratory analyses, overall recovery-pattern associations were observed for mechanical ventilation longer than 7 days (FDR-adjusted *p* = 0.002) and ICU stay longer than 14 days (FDR-adjusted *p* = 0.001); the ventilation estimates were non-monotonic across patterns, whereas all three non-reference patterns had higher odds of prolonged ICU stay. Adding the recovery pattern did not improve optimism-corrected discrimination (0.747 to 0.745; DeLong *p* = 0.365).

**Conclusion:**

Investigator-defined early marker recovery patterns described clinical heterogeneity but were not independently associated with 28-day mortality after geriatric adjustment and did not add predictive value. Exploratory overall associations with prolonged ventilation and ICU stay require external validation. Prospective evaluation is required before clinical use.

## 1 Introduction

Older adults now constitute a substantial proportion of patients treated in intensive care, and age alone provides little information about the likelihood or quality of recovery. Among survivors, critical illness can be followed by persistent functional dependence, cognitive impairment, and loss of independence, outcomes that are not adequately summarized by hospital survival alone (1–4). The clinical problem is therefore not simply to identify who is acutely ill at admission, but to recognize which patients fail to regain physiological stability after initial treatment.

Frailty is central to this distinction. It reflects diminished reserve across multiple organ systems and is conceptually different from chronological age or a single comorbidity count (5, 6). The Clinical Frailty Scale provides a pragmatic bedside estimate of baseline fitness and vulnerability (7). In ICU cohorts, frailty has repeatedly been associated with mortality, disability, institutional discharge, and impaired long-term quality of life (8–12). By contrast, APACHE II and SOFA primarily quantify acute physiological disturbance and organ failure (13, 14). These domains are complementary: acute severity describes the insult, whereas frailty and pre-illness function help describe the host in whom that insult occurs.

Inflammation and nutritional depletion provide another window into host response. Aging is accompanied by chronic low-grade inflammatory activation, and higher inflammatory burden is linked to frailty in community populations (15, 16). Critical illness can amplify this state through acute inflammatory activation, immune dysfunction, proteolysis, and catabolism (17). Commonly available laboratory indices reflect overlapping elements of this process. The C-reactive protein-to-albumin ratio (CAR) integrates an acute phase reactant with a negative acute-phase protein and has been associated with mortality in sepsis (18, 19). The neutrophil-to-lymphocyte ratio (NLR) captures leukocyte imbalance and has prognostic relevance in critical illness (20, 21). The systemic immune-inflammation index (SII) adds platelet count to neutrophil and lymphocyte information (22), while the prognostic nutritional index (PNI) combines albumin and lymphocyte count (23). Each marker is inexpensive, but none is biologically specific, and albumin in particular is influenced by inflammation, vascular permeability, dilution, and treatment as well as nutritional state (24).

Most prognostic studies evaluate these indices at a single time point. That approach cannot distinguish a patient who arrives with a high inflammatory burden but improves promptly from one whose abnormalities persist despite initial treatment. Serial C-reactive protein studies in infection and sepsis have shown that the direction of change may be more informative than the admission value alone (25–27). Nutritional risk is likewise relevant to early disability and recovery after critical illness (28, 29). However, combined early inflammatory and nutritional trajectories have been less well characterized in older, mixed ICU populations, particularly after accounting for frailty and comorbidity.

We therefore developed an investigator-defined framework based on changes in CAR, PNI, NLR, SII, and albumin between ICU admission and 48–72 h. Using a 72-h landmark design, we examined whether four operational recovery patterns were associated with 28-day mortality and other short-term outcomes. We also evaluated internal classification stability and whether the pattern added information beyond conventional clinical severity and geriatric risk. The analysis was exploratory and was not intended to establish a causal mechanism, universal cutoff, or treatment threshold.

## 2 Materials and methods

### 2.1 Study design and reporting

We conducted a single-center retrospective cohort study in the Geriatric Intensive Care Unit of the Fourth Affiliated Hospital of Nanjing Medical University from January 5, 2021, through May 31, 2026. The study was reported in accordance with the STROBE recommendations for observational research (30). Because the exposure required laboratory information through 72 h, follow-up began at a 72-h landmark (31), thereby avoiding assignment of exposure status during a period in which patients had to remain alive to be classified (32). Admission year was included to account for secular changes in case mix, laboratory practice, and ICU care across the study period.

### 2.2 Participants and cohort derivation

**Figure 1:**
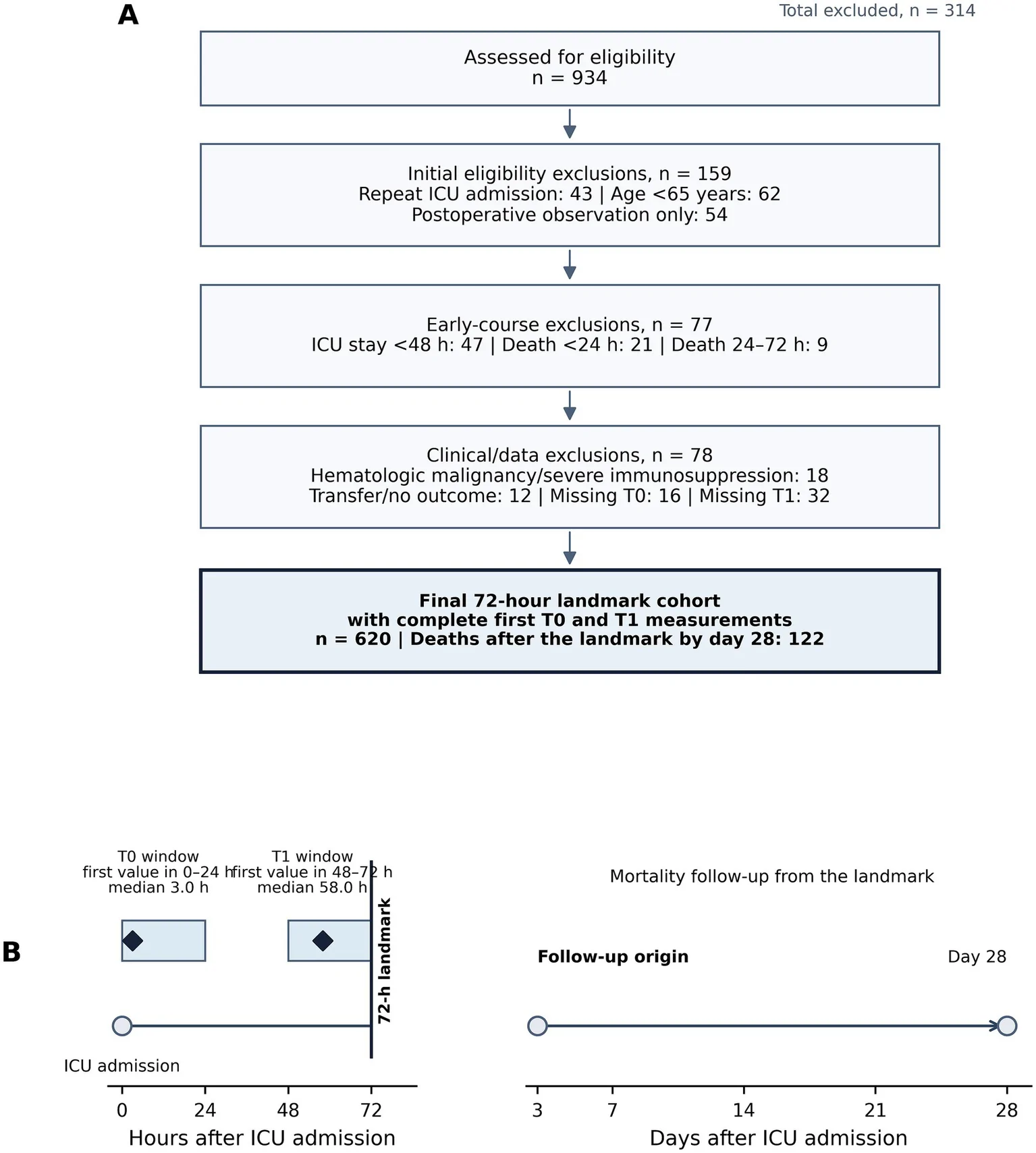
Patient selection and analysis timing. **(A)** Sequential derivation of the 72-h landmark cohort. **(B)** Separates the exposure-assignment window from outcome follow-up: *T0* was the first measurement within 0–24 h, *T1* was the first measurement within 48–72 h, and follow-up for 28-day mortality began at the 72-h landmark.

Admissions were eligible if the patient was aged 65 years or older and had a first ICU admission with outcome ascertainment. Sequential exclusions were repeat ICU admission, age younger than 65 years, postoperative observation without substantive critical care, ICU stay shorter than 48 h, death within 72 h, hematologic malignancy or severe immunosuppression, transfer with unavailable outcome, missing T0 core laboratory data, or missing T1 core laboratory measurements. The landmark cohort comprised patients alive beyond 72 h with complete first-window T0 and T1 measurements. Cohort derivation is shown in Figure 1 and Supplementary Table S3.

### 2.3 Clinical variables

Demographic and admission variables included age, sex, body mass index, admission source, and suspected infection documented by the treating ICU team at admission. Sepsis and septic shock represented diagnoses recorded at any point during the ICU stay rather than admission-only status. Respiratory failure was abstracted from the treating team’s clinical documentation. Acute illness severity was characterized using SOFA within 24 h and, when available, APACHE II (13, 14). Geriatric vulnerability was described using the Clinical Frailty Scale, pre-ICU Barthel Index, activities-of-daily-living dependency, pre-ICU bedridden status, dementia, dysphagia, and NRS-2002 (7, 28, 33). Comorbidity burden was summarized using the Charlson Comorbidity Index (34). Withdrawal/DNR/DNI denoted any documented treatment-withdrawal, do-not-resuscitate, or do-not-intubate decision during the index ICU stay.

The 9-level Clinical Frailty Scale was documented within 24 h of ICU admission and referenced health, activity, and function approximately 2 weeks before acute illness. Information was obtained from the patient when possible or otherwise from the primary caregiver and available records. Current intubation, bed confinement, or shock was not used to infer baseline frailty; CFS > =5 defined frailty. The 0–100 Barthel Index assessed 10 basic activities of daily living over the same premorbid period and was documented by a trained nurse or physician using a structured patient/caregiver interview and available records.

Invasive mechanical ventilation, vasopressor use, CRRT, albumin infusion, systemic corticosteroids, antibiotics, first-24-h fluid balance, and early enteral nutrition were recorded. Source-record review confirmed that positive treatment/support indicators began before T1 sampling, but exact patient-level start timestamps were not retained in the analytic extract. Pre-T1 treatment and support distributions are summarized in Supplementary Table S6.

### 2.4 Laboratory measurements and derived indices

Laboratory measurements were organized into T0 (0–24 h after ICU admission), T1 (48–72 h), and T2 (days 5–7). T0 and T1 were the first available measurements within their respective windows; when multiple measurements were available, values were not averaged and the worst value was not selected. The primary exposure used T0 and T1 only. T2 was used only for exploratory visualization because availability was affected by earlier death and discharge. NLR was calculated as neutrophils/lymphocytes, SII as platelets x neutrophils/lymphocytes, CAR as C-reactive protein (mg/L)/albumin (g/L), and PNI as albumin (g/L) + 5 x lymphocyte count (10^9/L). Absolute change was T1 minus T0, and relative change was (T1-T0)/T0 x 100%.

### 2.5 Operational recovery-pattern construction

Pattern assignment was performed without outcome information. Extreme marker status was defined using pooled T0-T1 internal quartiles: CAR >4.94, NLR > 19.40, or SII > 3281.7; PNI < =32.4 or albumin <=28.6 g/L. Improvement signals were CAR decrease > = 25%, NLR decrease > = 20%, SII decrease > = 20%, PNI increase > = 1.5, or albumin increase > = 0.5 g/L. Worsening signals were CAR increase > = 30%, NLR increase > = 25%, SII increase > = 25%, PNI decrease > = 1.5, or albumin decrease > = 0.8 g/L.

Pattern 1 required no T0 extreme marker, fewer than three worsening signals, and fewer than two T1 extreme markers. Pattern 2 required at least one T0 extreme marker, at least two improvement signals, and no worsening signal. Pattern 3 required at least one T0 extreme marker but did not fulfill the Pattern 2 improvement rule. Pattern 4 required no T0 extreme marker and either at least three worsening signals or at least two T1 extreme markers.

The thresholds and directional/count rules were finalized without reference to outcome status and applied uniformly. They are investigator-defined operational rules, not prospectively validated clinical cutoffs or data-derived biological patterns. Complete rules and executable code are provided in Supplementary Tables S1, S2 and the supplementary statistical materials.

### 2.6 Outcomes

The primary outcome was all-cause mortality by day 28 after ICU admission, analyzed from the 72-h landmark. Patients alive at day 28 were censored at that time. Exploratory secondary outcomes were ICU mortality, in-hospital mortality, mechanical ventilation longer than 7 days among invasively ventilated patients, ICU delirium among assessable patients, and ICU length of stay longer than 14 days.

RASS was recorded before CAM-ICU. RASS -4 or −5 denoted coma/deep unresponsiveness and was coded as unassessable rather than absence of delirium. RASS -3 to +4 triggered CAM-ICU assessment. Trained bedside ICU nurses assessed patients at least once per nursing shift, usually every 12 h, and after acute mental-status change; uncertain or conflicting records were reviewed by an intensivist. Delirium required at least one valid CAM-ICU-positive assessment, whereas a negative classification required at least one valid assessment and no positive assessment.

### 2.7 Statistical analysis

Continuous variables are reported as median and interquartile range and categorical variables as number and percentage, using non-missing observations as denominators. Recovery-pattern groups were compared using Kruskal–Wallis tests for continuous variables and chi-square, Fisher exact, or Monte Carlo conditional chi-square tests for categorical variables, as appropriate. These *p*-values were descriptive and were not used to select covariates.

Kaplan–Meier curves described survival from the 72-h landmark. Cox proportional-hazards models used Pattern 1 as the reference. Model 1 adjusted for age, sex, and admission year. Model 2 additionally included SOFA score and suspected infection at ICU admission. Model 3, the primary geriatric-adjusted model, further included the Clinical Frailty Scale and Charlson Comorbidity Index. SOFA was selected for the primary models because it was complete in the landmark cohort and directly represented early organ dysfunction; APACHE II, which was missing in 17 patients, replaced SOFA in a sensitivity analysis. ICU-wide sepsis, mechanical ventilation, vasopressor use, and CRRT were not treated as admission covariates because they could occur during pattern formation. An extended ICU-course covariate model additionally including these variables was evaluated as a sensitivity analysis. Proportional hazards were assessed using Schoenfeld residuals, and the four-level pattern was tested jointly.

For each of the five exploratory secondary outcomes, logistic regression included all four recovery patterns and adjusted for age, sex, admission year, SOFA score, suspected infection at ICU admission, Clinical Frailty Scale, and Charlson Comorbidity Index. The prolonged-ventilation model was restricted to invasively ventilated patients, and the delirium model to patients with at least one valid CAM-ICU assessment. A 3-degree-of-freedom likelihood-ratio test evaluated the overall recovery-pattern term for each outcome. Benjamini-Hochberg correction controlled the false discovery rate across the five overall secondary-outcome tests only (35). Pattern-specific odds ratios versus Pattern 1 characterized the form of any overall association; these contrasts were exploratory and were not separately adjusted for multiplicity. Descriptive outcomes for all four patterns are reported in the Supplementary material.

The primary geriatric-adjusted model used complete cases. Multiple imputation by chained equations was performed in 20 datasets as a sensitivity analysis; the event indicator and Nelson-Aalen cumulative hazard were included, and imputed Clinical Frailty Scale values were rounded and constrained to 1–9 (36). Additional sensitivity analyses evaluated the extended ICU-course covariate model, adjusted for pre-T1 treatment and organ support, excluded withdrawal/DNR/DNI, albumin infusion, or systemic corticosteroid exposure, replaced SOFA with APACHE II, used T0-only thresholds, modeled admission year categorically, evaluated continuous markers, and perturbed the directional and count rules.

Internal bootstrap assessment used 1,000 samples. Within each sample, pooled marker thresholds were re-estimated, patients were reassigned using the fixed directional and count rules, and the Cox and prediction models were refitted. Exploratory prediction models sequentially included clinical variables, geriatric variables, and the recovery pattern. Discrimination, Brier score, calibration intercept and slope, and decision-curve net benefit were assessed; optimism was estimated by bootstrap (37), paired AUROCs were compared by the DeLong method, and likelihood-ratio tests evaluated incremental model fit. Decision-curve analysis was interpreted only as exploratory (38), and small AUROC changes were not treated as evidence of clinical utility (39). All tests were two-sided. Analyses were performed in Python.

### 2.8 Ethics statement

The study was approved by the Ethics Committee of the Fourth Affiliated Hospital of Nanjing Medical University (approval no. nmd12036). The requirement for written informed consent was waived because the study retrospectively analyzed de-identified clinical data.

## 3 Results

### 3.1 Cohort derivation and data completeness

Of 934 ICU admissions assessed for eligibility, 314 were excluded sequentially. The most frequent exclusions were age younger than 65 years (*n* = 62), postoperative observation only (*n* = 54), ICU stay shorter than 48 h (*n* = 47), repeat ICU admission (*n* = 43), and missing T1 core measurements (*n* = 32). The final 72-h landmark cohort included 620 patients; 122 died after the landmark and before day 28 (Figure 1 and Supplementary Table S3).

T0 and T1 core markers and the primary outcome were complete. Median sampling times were 3.0 h (IQR, 1.9–4.6) for T0 and 58.0 h (IQR, 54.0–62.1) for T1. The Clinical Frailty Scale was missing in 24 patients (3.9%), leaving 596 patients and 119 events in the primary complete-case model. Missingness was 8.7% for the pre-ICU Barthel Index/ADL dependency and 12.4% for NRS-2002. T2 markers were available for 372 patients (60.0%) and were restricted to exploratory visualization (Supplementary Tables S4, S5 and Supplementary Figure S5).

### 3.2 Baseline characteristics and early trajectories

The median age was 78 years (IQR, 73–83), and 329 patients (53.1%) were men. Pattern 1 (no initial extreme burden without delayed-deterioration-rule fulfillment) included 255 patients (41.1%), Pattern 2 (initial extreme burden with improvement-rule fulfillment) 114 (18.4%), Pattern 3 (initial extreme burden without improvement-rule fulfillment) 180 (29.0%), and Pattern 4 (no initial extreme burden with delayed-deterioration-rule fulfillment) 71 (11.5%). Pattern 3 had greater acute severity and geriatric vulnerability: median SOFA was 8 (IQR, 6–9), 60.6% had sepsis during the ICU stay, and 63.1% of patients with available CFS data had CFS > =5 (Table 1). Group-comparison *p*-values were descriptive.

**Table 1:** Baseline characteristics by operational recovery pattern.

Characteristic	Overall	Missing n	Pattern 1	Pattern 2	Pattern 3	Pattern 4	*P*-value
Age, years	78.0 [73.0, 83.0]	0	77.0 [72.5, 82.0]	77.0 [74.0, 83.0]	79.0 [75.0, 84.0]	78.0 [73.5, 83.0]	0.012
Male sex	329 (53.1%)	0	140 (54.9%)	56 (49.1%)	96 (53.3%)	37 (52.1%)	0.780
Body mass index, kg/m^2^	23.4 [21.3, 25.6]	38	23.8 [21.6, 25.9]	22.8 [20.8, 25.4]	23.3 [20.6, 25.2]	23.7 [21.7, 25.8]	0.255
Admission source		0					0.532
Emergency department	265 (42.7%)		108 (42.4%)	48 (42.1%)	79 (43.9%)	30 (42.3%)	
Outside hospital	89 (14.4%)		37 (14.5%)	16 (14.0%)	21 (11.7%)	15 (21.1%)	
Operating room	63 (10.2%)		27 (10.6%)	7 (6.1%)	24 (13.3%)	5 (7.0%)	
Hospital ward	181 (29.2%)		72 (28.2%)	40 (35.1%)	49 (27.2%)	20 (28.2%)	
Nursing facility	22 (3.5%)		11 (4.3%)	3 (2.6%)	7 (3.9%)	1 (1.4%)	
Suspected infection at admission	395 (63.7%)	0	145 (56.9%)	80 (70.2%)	127 (70.6%)	43 (60.6%)	0.011
Sepsis during ICU stay	234 (37.7%)	0	54 (21.2%)	65 (57.0%)	109 (60.6%)	6 (8.5%)	<0.001
Septic shock during ICU stay	121 (19.5%)	0	17 (6.7%)	35 (30.7%)	68 (37.8%)	1 (1.4%)	<0.001
Respiratory failure	341 (55.0%)	0	125 (49.0%)	77 (67.5%)	103 (57.2%)	36 (50.7%)	0.008
SOFA score within 24 h	6.0 [5.0, 8.0]	0	5.0 [4.0, 7.0]	7.0 [5.0, 9.0]	8.0 [6.0, 9.0]	6.0 [4.0, 7.0]	<0.001
APACHE II score within 24 h	20.0 [17.0, 23.0]	17	18.0 [16.0, 21.0]	21.0 [18.0, 23.0]	21.0 [19.0, 25.8]	19.0 [16.2, 22.0]	<0.001
Clinical Frailty Scale	5.0 [4.0, 5.0]	24	4.0 [3.0, 5.0]	5.0 [4.0, 6.0]	5.0 [4.0, 6.0]	4.0 [4.0, 5.0]	<0.001
Frailty, CFS > =5	301 (50.5%)	24	93 (38.3%)	65 (60.2%)	111 (63.1%)	32 (46.4%)	<0.001
Pre-ICU Barthel Index	70.0 [50.0, 85.0]	54	75.0 [60.0, 90.0]	60.0 [45.0, 80.0]	62.5 [40.0, 80.0]	70.0 [45.0, 86.2]	<0.001
ADL dependency		54					<0.001
Independent/minimal dependence	213 (37.6%)		116 (48.9%)	27 (26.7%)	47 (28.7%)	23 (35.9%)	
Mild dependence	155 (27.4%)		63 (26.6%)	29 (28.7%)	48 (29.3%)	15 (23.4%)	
Moderate dependence	107 (18.9%)		37 (15.6%)	26 (25.7%)	30 (18.3%)	14 (21.9%)	
Severe/total dependence	91 (16.1%)		21 (8.9%)	19 (18.8%)	39 (23.8%)	12 (18.8%)	
Pre-ICU bedridden status	185 (29.8%)	0	48 (18.8%)	45 (39.5%)	66 (36.7%)	26 (36.6%)	<0.001
Dementia	109 (17.6%)	0	32 (12.5%)	25 (21.9%)	40 (22.2%)	12 (16.9%)	0.034
Dysphagia	143 (23.1%)	0	44 (17.3%)	30 (26.3%)	51 (28.3%)	18 (25.4%)	0.036
NRS-2002 score	2.0 [2.0, 3.0]	77	2.0 [1.0, 3.0]	3.0 [2.0, 3.0]	3.0 [2.0, 4.0]	2.0 [1.8, 3.0]	<0.001
Nutritional risk, NRS-2002 > =3	246 (45.3%)	77	76 (34.1%)	56 (58.9%)	93 (59.2%)	21 (30.9%)	<0.001
Charlson Comorbidity Index	6.0 [4.0, 7.0]	0	5.0 [4.0, 7.0]	6.0 [4.0, 7.0]	6.0 [5.0, 8.0]	5.0 [4.0, 7.0]	0.005
Pre-T1 invasive mechanical ventilation	363 (58.5%)	0	131 (51.4%)	80 (70.2%)	120 (66.7%)	32 (45.1%)	<0.001
Pre-T1 vasopressor use	249 (40.2%)	0	63 (24.7%)	61 (53.5%)	105 (58.3%)	20 (28.2%)	<0.001
Pre-T1 CRRT use	86 (13.9%)	0	21 (8.2%)	22 (19.3%)	35 (19.4%)	8 (11.3%)	0.002
Pre-T1 albumin infusion	157 (25.3%)	0	44 (17.3%)	43 (37.7%)	57 (31.7%)	13 (18.3%)	<0.001
Withdrawal or DNR/DNI	59 (9.5%)	0	20 (7.8%)	13 (11.4%)	21 (11.7%)	5 (7.0%)	0.428

Continuous variables are median [IQR], and categorical variables are *n* (%), using non-missing observations as denominators. Missingness is reported by variable. *P*-values are descriptive and were not used for covariate selection. Pattern 1, no initial extreme burden without delayed-deterioration-rule fulfillment; Pattern 2, initial extreme burden with improvement-rule fulfillment; Pattern 3, initial extreme burden without improvement-rule fulfillment; Pattern 4, no initial extreme burden with delayed-deterioration-rule fulfillment. ADL, activities of daily living; APACHE II, Acute Physiology and Chronic Health Evaluation II; CFS, Clinical Frailty Scale; CRRT, continuous renal replacement therapy; DNI, do-not-intubate; DNR, do-not-resuscitate; ICU, intensive care unit; NRS-2002, Nutritional Risk Screening 2002; SOFA, Sequential Organ Failure Assessment. Pre-T1 treatment and support indicators were confirmed by source-record review to have begun before T1 sampling; exact patient-level start timestamps were not retained in the analytic extract.

**Figure 2:**
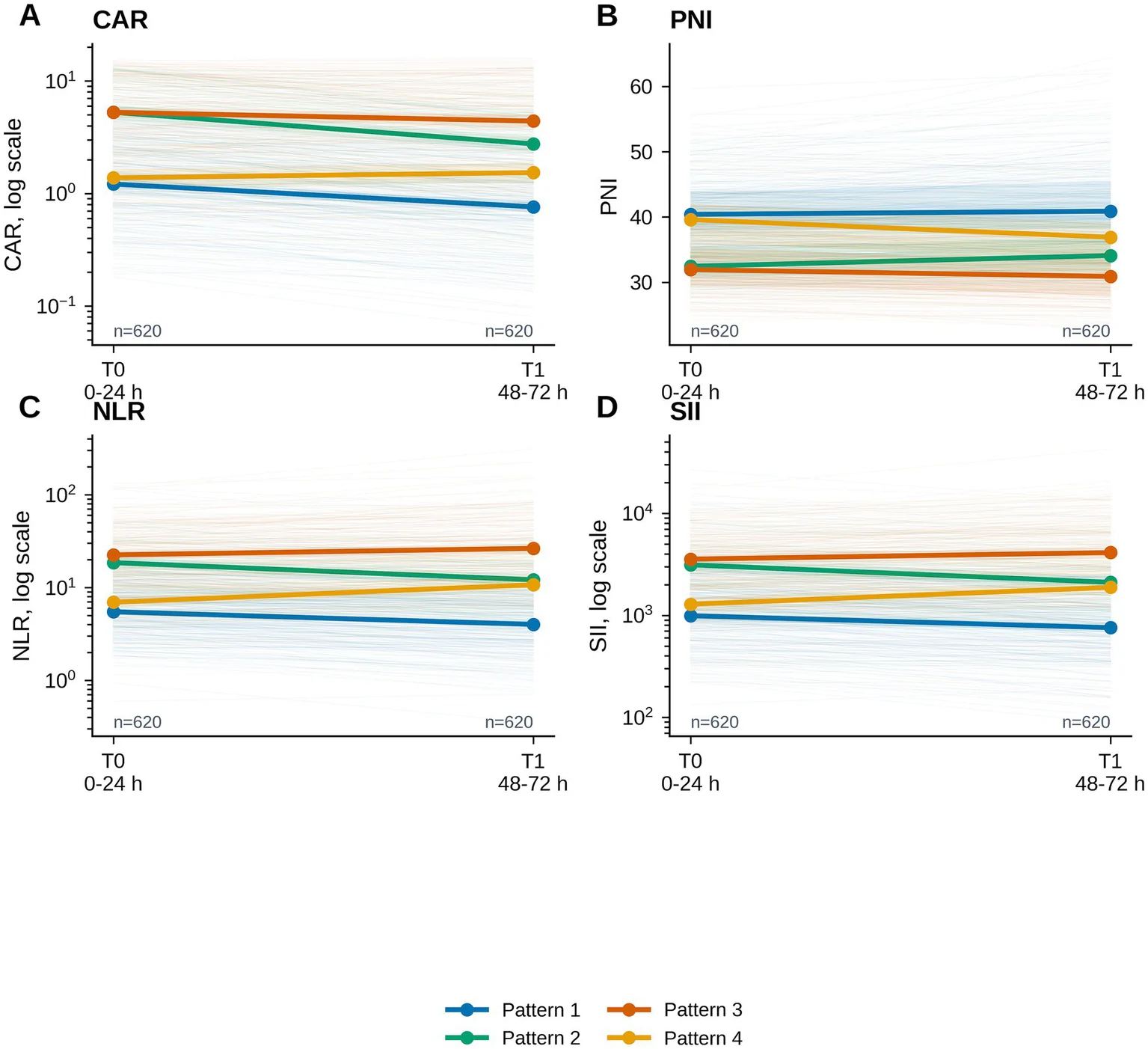
T0–T1 inflammatory and nutritional marker trajectories by operational recovery pattern. Thin lines represent individual patients. Bold lines and ribbons show geometric means and 95% confidence intervals for CAR, NLR, and SII on logarithmic axes and medians and interquartile ranges for PNI. The displayed *n* = 620 at each time point refers to the full cohort; group sizes were 255, 114, 180, and 71 for Patterns 1–4, respectively. T2 was not used for category construction and is shown only in Supplementary Figure S5. CAR, C-reactive protein-to-albumin ratio; NLR, neutrophil-to-lymphocyte ratio; PNI, prognostic nutritional index; SII, systemic immune-inflammation index. **(A)** CAR, **(B)** PNI, **(C)** NLR, and **(D)** SII.

Marker trajectories differed by pattern (Table 2 and Figure 2). At T1, Pattern 3 had median CRP 135.4 mg/L (IQR, 76.8–245.0), CAR 5.1 (2.7–8.5), NLR 26.2 (14.6–48.0), SII 4221.8 (2541.3–7327.2), albumin 27.8 g/L (25.8–30.2), and PNI 30.9 (28.0–33.5). Pattern 2 showed median reductions of 49.3% in CAR, 32.4% in NLR, and 31.0% in SII, with a 3.6% increase in PNI. Pattern 4 showed median increases of 50.2% in NLR and 43.7% in SII and a 7.5% decrease in PNI. Marker distributions and correlations are shown in Supplementary Figures S1, S2.

**Table 2:** T0-T1 laboratory profiles and changes by operational recovery pattern.

Characteristic	Overall	Missing n	Pattern 1	Pattern 2	Pattern 3	Pattern 4	*P*-value
CRP at T0, mg/L	76.2 [37.7, 168.5]	0	42.8 [24.3, 73.8]	181.9 [86.7, 315.4]	173.9 [87.9, 321.6]	47.6 [26.1, 78.3]	<0.001
CRP at T1, mg/L	54.2 [25.1, 127.5]	0	26.7 [15.2, 46.6]	98.2 [43.0, 153.1]	135.4 [76.8, 245.0]	50.7 [26.6, 95.2]	<0.001
Albumin at T0, g/L	31.3 [28.7, 33.7]	0	33.3 [31.3, 35.2]	28.6 [27.4, 31.4]	28.5 [26.8, 30.9]	32.8 [31.0, 34.3]	<0.001
Albumin at T1, g/L	31.2 [28.5, 33.9]	0	33.6 [31.4, 35.5]	29.5 [28.3, 32.0]	27.8 [25.8, 30.2]	32.0 [29.8, 33.7]	<0.001
CAR at T0	2.4 [1.1, 5.7]	0	1.3 [0.7, 2.3]	6.2 [3.0, 10.2]	5.7 [3.1, 10.8]	1.3 [0.8, 2.4]	<0.001
CAR at T1	1.8 [0.8, 4.4]	0	0.8 [0.5, 1.4]	3.1 [1.4, 5.0]	5.1 [2.7, 8.5]	1.5 [0.8, 3.1]	<0.001
PNI at T0	36.7 [32.6, 40.9]	0	40.4 [38.0, 43.8]	32.5 [30.8, 35.5]	31.9 [29.6, 35.2]	39.6 [36.3, 42.1]	<0.001
PNI at T1	36.2 [32.1, 40.8]	0	40.9 [38.3, 45.5]	34.1 [31.9, 36.9]	30.9 [28.0, 33.5]	36.9 [32.8, 39.0]	<0.001
NLR at T0	10.4 [5.4, 19.5]	0	5.7 [3.6, 8.6]	18.6 [11.6, 28.1]	23.6 [13.6, 35.8]	7.6 [4.8, 10.3]	<0.001
NLR at T1	9.1 [4.4, 19.2]	0	4.2 [2.5, 6.8]	11.9 [7.3, 19.1]	26.2 [14.6, 48.0]	12.0 [6.9, 16.3]	<0.001
SII at T0	1852.8 [1005.9, 3295.7]	0	1024.9 [628.0, 1603.1]	3159.4 [1981.9, 4691.9]	3831.8 [2185.2, 5781.2]	1410.2 [896.4, 2013.8]	<0.001
SII at T1	1665.6 [826.5, 3260.4]	0	826.0 [464.1, 1270.3]	2087.1 [1149.9, 3439.1]	4221.8 [2541.3, 7327.2]	2138.4 [1299.7, 3068.5]	<0.001
Delta CAR, T1-T0	−0.5 [−1.6, −0.0]	0	−0.3 [−0.9, −0.1]	−2.3 [−4.5, −1.0]	−0.5 [−2.2, 0.7]	0.2 [−0.3, 0.8]	<0.001
Relative delta CAR, %	−29.9 [−51.2, −0.4]	0	−36.4 [−55.0, −12.2]	−49.3 [−59.3, −35.1]	−12.2 [−41.2, 12.0]	6.1 [−25.6, 63.3]	<0.001
Delta PNI, T1-T0	−0.3 [−1.9, 1.3]	0	0.6 [−0.9, 2.3]	1.2 [0.5, 2.4]	−1.4 [−2.2, −0.3]	−2.9 [−4.1, −2.3]	<0.001
Relative delta PNI, %	−0.8 [−5.8, 3.6]	0	1.5 [−2.3, 5.7]	3.6 [1.5, 7.7]	−4.5 [−7.1, −0.9]	−7.5 [−9.3, −6.0]	<0.001
Delta NLR, T1-T0	−0.7 [−3.3, 2.4]	0	−1.2 [−2.7, −0.3]	−5.6 [−9.7, −2.2]	3.1 [−1.3, 12.4]	3.8 [2.1, 5.9]	<0.001
Relative delta NLR, %	−10.5 [−34.6, 23.5]	0	−25.4 [−42.9, −7.2]	−32.4 [−49.4, −16.0]	16.0 [−6.7, 50.2]	50.2 [35.6, 74.5]	<0.001
Delta SII, T1-T0	−93.4 [−558.3, 420.1]	0	−162.1 [−423.8, 23.2]	−824.0 [−1513.7, −321.3]	502.7 [−340.2, 2024.9]	555.4 [284.7, 1039.6]	<0.001
Relative delta SII, %	−8.1 [−33.4, 25.4]	0	−23.0 [−40.8, 1.8]	−31.0 [−42.3, −14.0]	21.5 [−11.1, 53.0]	43.7 [19.0, 68.6]	<0.001

Values are median [IQR]. T0 was the first measurement within 0–24 h after ICU admission; T1 was the first measurement within 48–72 h. CAR, C-reactive protein-to-albumin ratio; CRP, C-reactive protein; NLR, neutrophil-to-lymphocyte ratio; PNI, prognostic nutritional index; SII, systemic immune-inflammation index.

### 3.3 Operational recovery patterns and 28-day mortality

**Figure 3:**
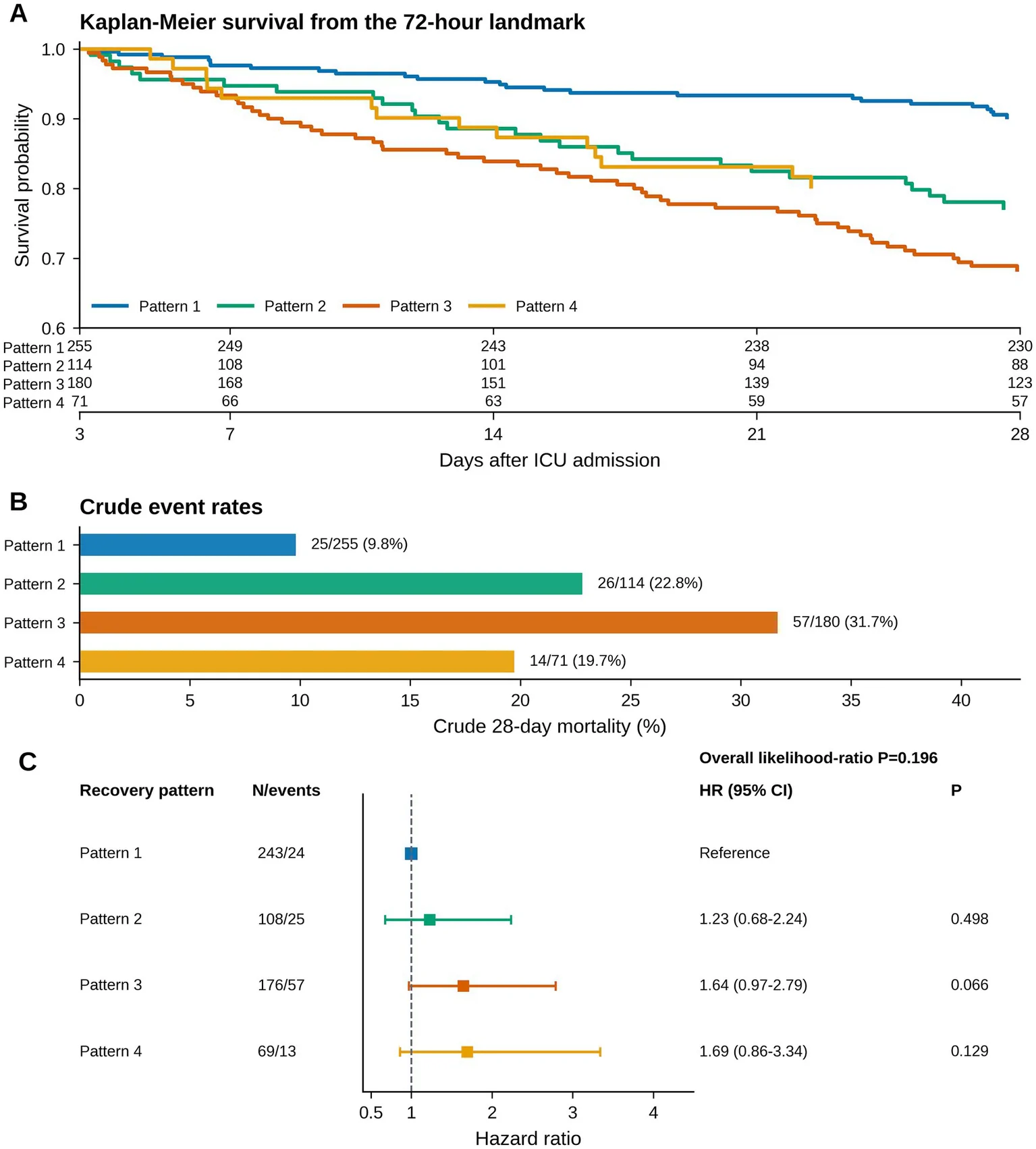
Mortality risk by operational recovery pattern. Panel **(A)** shows Kaplan–Meier survival from the 72-h landmark to day 28 with numbers at risk. Panel **(B)** shows crude event rates in all 620 patients. Panel **(C)** shows hazard ratios in 596 complete cases from the geriatric-adjusted primary Cox model, adjusted for age, sex, admission year, SOFA score, suspected infection at ICU admission, Clinical Frailty Scale, and Charlson Comorbidity Index. Pattern 1 is the reference. The overall four-level likelihood-ratio *p*-value is displayed once. CI, confidence interval; HR, hazard ratio; SOFA, Sequential Organ Failure Assessment.

Crude 28-day mortality was 9.8% (25/255) in Pattern 1, 22.8% (26/114) in Pattern 2, 31.7% (57/180) in Pattern 3, and 19.7% (14/71) in Pattern 4 (Figure 3).

The recovery pattern was associated with mortality in the demographic model (overall *p* < 0.001) and baseline clinical-severity model (overall *p* = 0.009), but not in the geriatric-adjusted primary model (overall likelihood-ratio *p* = 0.196). In the primary model, none of the non-reference patterns reached statistical significance: Pattern 2, HR 1.23 (95% CI, 0.68–2.24; *p* = 0.498); Pattern 3, HR 1.64 (95% CI, 0.97–2.79; *p* = 0.066); and Pattern 4, HR 1.69 (95% CI, 0.86–3.34; *p* = 0.129) (Table 3 and Figure 3). The proportional-hazards assumption was not rejected (global *p* = 0.548; Supplementary Table S12).

**Table 3:** Association between operational recovery pattern and 28-day mortality.

Model	Pattern	*N*	Events	HR (95% CI)	*P*-value	Overall LR P
Model 1: demographics	Pattern 2	620	122	2.52 (1.46–4.37)	<0.001	<0.001
	Pattern 3	620	122	3.35 (2.09–5.38)	<0.001	
	Pattern 4	620	122	2.10 (1.09–4.03)	0.027	
Model 2: baseline clinical severity	Pattern 2	620	122	1.73 (0.97–3.07)	0.063	0.009
	Pattern 3	620	122	2.26 (1.37–3.74)	0.001	
	Pattern 4	620	122	2.05 (1.07–3.96)	0.031	
Model 3: geriatric-adjusted primary model	Pattern 2	596	119	1.23 (0.68–2.24)	0.498	0.196
	Pattern 3	596	119	1.64 (0.97–2.79)	0.066	
	Pattern 4	596	119	1.69 (0.86–3.34)	0.129	

Pattern 1 is the reference. Model 1 adjusted for age, sex, and admission year. Model 2 additionally adjusted for SOFA score and suspected infection at ICU admission. Model 3, the primary model, additionally adjusted for Clinical Frailty Scale and Charlson Comorbidity Index. The overall likelihood-ratio *P*-value tests the three pattern indicators jointly. HR, hazard ratio; SOFA, Sequential Organ Failure Assessment.

### 3.4 Exploratory clinical outcomes

**Figure 4:**
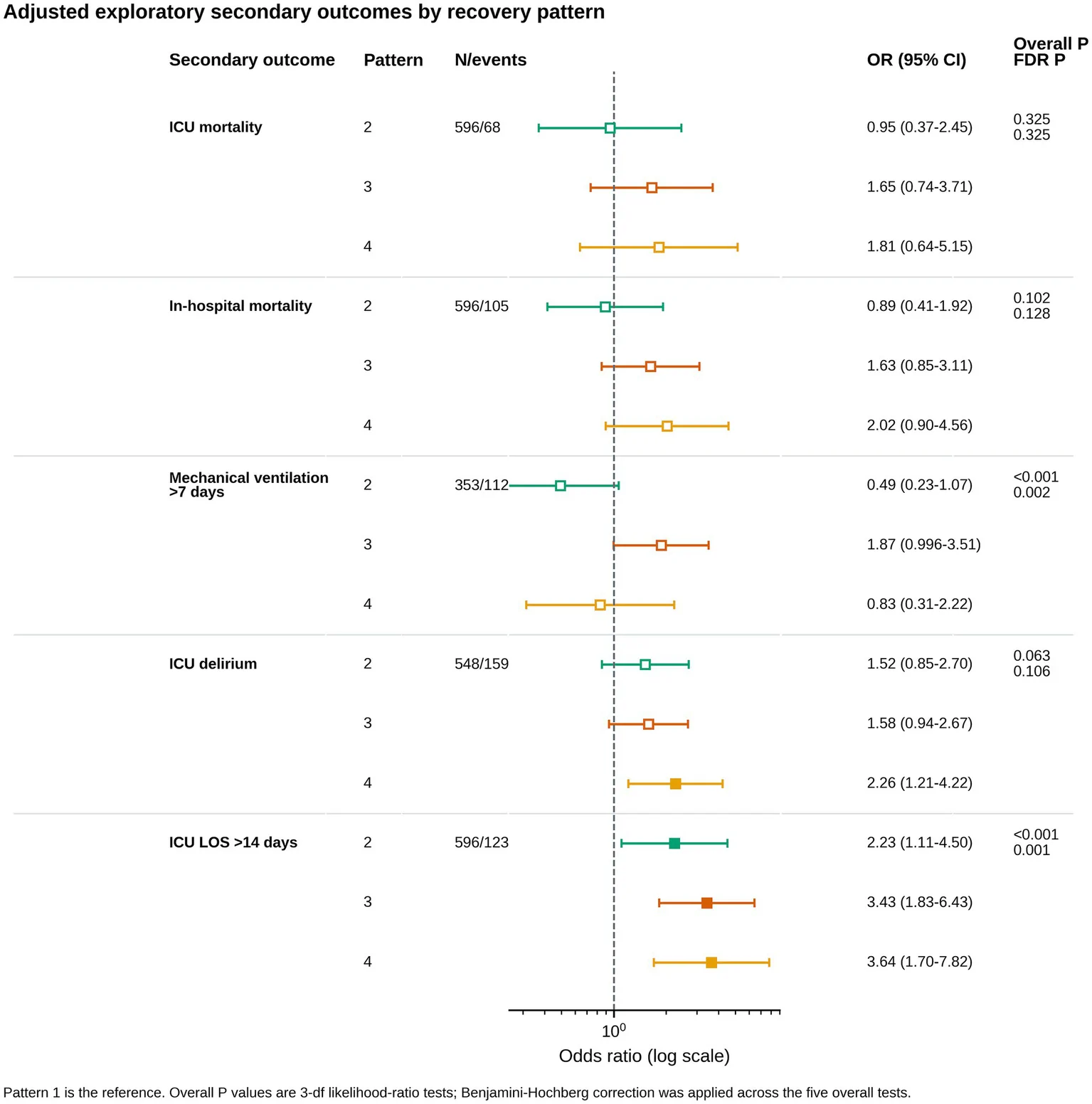
Adjusted exploratory secondary outcomes by recovery pattern. Pattern 1 was the reference. Odds ratios were estimated using logistic regression adjusted for age, sex, admission year, SOFA score, suspected infection at ICU admission, Clinical Frailty Scale, and Charlson Comorbidity Index. The prolonged-ventilation analysis was restricted to invasively ventilated patients and the delirium analysis to patients with at least one valid CAM-ICU assessment. For each outcome, the overall *p*-value is from a 3-degree-of-freedom likelihood-ratio test of the three recovery-pattern indicators; Benjamini–Hochberg correction was applied across the five overall tests. Pattern-specific *p*-values were exploratory and were not separately adjusted for multiplicity. Filled squares denote unadjusted pattern-specific *p* < 0.05. CI, confidence interval; FDR, false discovery rate; ICU, intensive care unit; LOS, length of stay; OR, odds ratio.

Overall recovery-pattern associations were observed for mechanical ventilation longer than 7 days (likelihood-ratio *p* < 0.001; FDR-adjusted *p* = 0.002) and ICU length of stay longer than 14 days (likelihood-ratio *p* < 0.001; FDR-adjusted *p* = 0.001). The prolonged-ventilation estimates were non-monotonic: ORs versus Pattern 1 were 0.49 (95% CI, 0.23–1.07) for Pattern 2, 1.87 (95% CI, 0.996–3.51) for Pattern 3, and 0.83 (95% CI, 0.31–2.22) for Pattern 4. For ICU length of stay longer than 14 days, the corresponding ORs were 2.23 (95% CI, 1.11–4.50), 3.43 (95% CI, 1.83–6.43), and 3.64 (95% CI, 1.70–7.82). Overall tests were not significant after FDR correction for ICU mortality, in-hospital mortality, or ICU delirium (Table 4 and Figure 4). Descriptive and adjusted results for all four patterns are reported in Supplementary Tables S7, S8.

**Table 4:** Adjusted exploratory secondary outcomes by recovery pattern.

Outcome	N/events	Pattern	OR (95% CI)	*P*-value	Overall LR P	Overall FDR P
ICU mortality	596/68	Pattern 2	0.95 (0.37–2.45)	0.918	0.325	0.325
		Pattern 3	1.65 (0.74–3.71)	0.223		
		Pattern 4	1.81 (0.64–5.15)	0.263		
In-hospital mortality	596/105	Pattern 2	0.89 (0.41–1.92)	0.770	0.102	0.128
		Pattern 3	1.63 (0.85–3.11)	0.142		
		Pattern 4	2.02 (0.90–4.56)	0.089		
Mechanical ventilation >7 days	353/112	Pattern 2	0.49 (0.23–1.07)	0.072	<0.001	0.002
		Pattern 3	1.87 (0.996–3.51)	0.052		
		Pattern 4	0.83 (0.31–2.22)	0.717		
ICU delirium	548/159	Pattern 2	1.52 (0.85–2.70)	0.156	0.063	0.106
		Pattern 3	1.58 (0.94–2.67)	0.086		
		Pattern 4	2.26 (1.21–4.22)	0.010		
ICU LOS > 14 days	596/123	Pattern 2	2.23 (1.11–4.50)	0.025	<0.001	0.001
		Pattern 3	3.43 (1.83–6.43)	<0.001		
		Pattern 4	3.64 (1.70–7.82)	<0.001		

Pattern 1 is the reference. All four patterns were included in each logistic model. Models adjusted for age, sex, admission year, SOFA score, suspected infection at ICU admission, Clinical Frailty Scale, and Charlson Comorbidity Index. The prolonged-ventilation analysis was restricted to invasively ventilated patients, and the delirium analysis to patients with at least one valid CAM-ICU assessment. Overall *P*-values are 3-degree-of-freedom likelihood-ratio tests of the three recovery-pattern indicators. Benjamini–Hochberg correction was applied across the five overall tests; pattern-specific *p*-values were not separately adjusted for multiplicity. FDR, false discovery rate; ICU, intensive care unit; LOS, length of stay; OR, odds ratio; SOFA, Sequential Organ Failure Assessment.

### 3.5 Sensitivity and exploratory prediction analyses

Sensitivity analyses showed consistent effect direction but model-dependent statistical significance. The Pattern 3 estimate was HR 1.63 (95% CI, 0.95–2.79; *p* = 0.078) under the extended ICU-course covariate model and HR 1.62 (95% CI, 0.96–2.75; *p* = 0.071) with T0-only thresholds. Multiple imputation yielded HR 1.79 (95% CI, 1.06–3.01; *p* = 0.029), APACHE II replacement of SOFA yielded HR 2.09 (95% CI, 1.25–3.48; *p* = 0.005), and exclusion of pre-T1 corticosteroid exposure yielded HR 2.07 (95% CI, 1.08–3.98; *p* = 0.029). Across 12 directional/count-rule perturbations, Pattern 3 hazard ratios ranged from 1.33 to 1.64 and all *p* values ranged from 0.066 to 0.271 (Supplementary Tables S9, S10).

In 1,000 bootstrap samples with threshold re-estimation, reclassification, and model refitting, median classification agreement was 0.984 (95% interval, 0.958–0.995) and median Cohen kappa was 0.977 (0.941–0.993). Twenty-four patients had reference-pattern stability below 0.80, indicating boundary sensitivity in a small subset (Supplementary Table S11 and Supplementary Figure S4). In prediction analyses, the optimism-corrected AUROC was 0.717 for the clinical model, 0.747 after adding geriatric variables, and 0.745 after adding the recovery pattern. The recovery pattern did not improve discrimination (DeLong *p* = 0.365) or model fit (likelihood-ratio *p* = 0.231) (Supplementary Table S13 and Supplementary Figure S3).

## 4 Discussion

This 72-h landmark study evaluated an investigator-defined, rule-based description of early inflammatory and nutritional marker recovery in older ICU patients. The four patterns differed markedly in acute severity, frailty, marker trajectories, and crude outcomes. However, after geriatric adjustment, the four-level pattern was not independently associated with 28-day mortality, and none of the three non-reference comparisons was statistically significant. The pattern also failed to improve optimism-corrected discrimination. Exploratory overall associations were observed for prolonged ventilation and prolonged ICU stay. The ventilation estimates were non-monotonic across patterns, whereas all three non-reference patterns had higher odds of prolonged ICU stay. These findings support descriptive risk characterization, not a validated mortality score, biological phenotype, or treatment rule.

The distinction between Patterns 2 and 3 illustrates the purpose of a trajectory-based description. Both categories began with at least one extreme marker, but only Pattern 2 fulfilled the operational improvement rule. Serial CRP studies similarly distinguish declining concentrations from persistently high or rising values (25, 26), and a more recent sepsis study identified persistently high CRP trajectories as the group with the highest in-hospital mortality (27). This trajectory perspective differs from studies based on admission CAR (18, 19) or NLR (21) alone. Pattern 4 likewise shows that an initially lower-burden profile can deteriorate by 48–72 h. These distinctions support descriptive reassessment but do not validate the categories as biological patterns.

The composite indices should be interpreted as summaries of an observed clinical state rather than discrete biological pathways. CAR combines CRP with albumin, which is affected by inflammation, capillary leakage, distribution volume, catabolism, and exogenous administration (24). NLR reflects stress leukocyte redistribution as well as infection, corticosteroid exposure, and immune suppression (20). SII reuses neutrophil and lymphocyte counts and adds platelets, whereas PNI reuses albumin and lymphocytes (22, 23). The strong cross-marker correlations were therefore expected, and the term inflammatory and nutritional marker pattern is more accurate than a mechanistic inflammatory and nutritional marker phenotype.

The change after geriatric adjustment is clinically informative. Frailty represents diminished resilience to acute stress and is associated with mortality and failure to return home after critical illness (6, 8, 10). Among survivors, it is also associated with disability and impaired health-related quality of life (9, 40). The VIP2 study showed that frailty, cognition, activities of daily living, and comorbidity make distinct contributions to outcome in very old ICU patients (12), while chronic inflammation is associated with frailty (15, 16). In our analysis, the Pattern 3 hazard ratio decreased from 2.26 in the baseline clinical-severity model to 1.64 in the geriatric-adjusted model, and the global pattern *p* value changed from 0.009 to 0.196. This change may reflect confounding or shared vulnerability and cannot be interpreted as causal mediation.

The clearest exploratory finding involved ICU length of stay. All three non-reference patterns had higher adjusted odds of ICU stay longer than 14 days, with the largest estimates for Patterns 3 and 4. For Pattern 3, the direction was maintained in ICU-survivor, day-28-survivor, continuous-length-of-stay, and live-discharge analyses (Supplementary Table S14). Persistent inflammation, immunosuppression, and catabolism have been described in chronic critical illness (17), and early quadriceps muscle loss is related to frailty, nutritional risk, and mortality (41). Poor nutritional status has also been associated with adverse outcomes in older ventilated patients (42), and admission nutritional risk with institutional discharge and early disability among survivors (29). Our data do not establish persistent inflammation, immunosuppression, and catabolism syndrome, but they suggest that both initial extreme burden and delayed deterioration may accompany a more prolonged ICU course.

An overall association was also observed for mechanical ventilation longer than 7 days, but the pattern-specific estimates were non-monotonic: lower for Pattern 2, higher for Pattern 3, and near null for Pattern 4. Because this analysis was restricted to patients receiving invasive ventilation, it may reflect heterogeneity in ventilation indication, treatment response, extubation readiness, and the competing processes of death and liberation from ventilation. It should be considered hypothesis-generating rather than evidence of an ordered recovery gradient.

The overall recovery-pattern test for ICU delirium did not remain significant after false-discovery-rate correction, although the unadjusted Pattern 4-versus-Pattern 1 contrast was positive. Because pattern-specific contrasts were not separately adjusted for multiplicity, this finding is hypothesis-generating. Delirium is common during critical illness and is associated with mortality, longer hospitalization, and subsequent cognitive impairment (4, 43, 44). Illness severity, sedative exposure, ventilation, and pre-existing cognitive vulnerability overlap with the variables represented by the recovery patterns, so residual confounding remains likely. These findings do not imply that modifying the laboratory profile would prevent delirium.

The prediction analyses further support restraint. Adding geriatric variables increased the apparent AUROC from 0.730 to 0.761. Adding the recovery pattern increased the apparent AUROC to 0.767, but the optimism-corrected AUROC changed from 0.747 to 0.745; neither the DeLong comparison (*p* = 0.365) nor the likelihood-ratio test (*p* = 0.231) supported incremental value. Small AUROC changes can be misleading when baseline discrimination is already moderate (39), and internal bootstrap correction cannot substitute for temporal or external validation (37). Decision-curve analysis is meaningful only when thresholds and subsequent actions are clinically defensible (38). No pattern-directed action was tested here.

A practical implication is that 48–72 h may be a reasonable time for structured reassessment rather than automatic intervention. At this point clinicians reconsider infection control, organ support, fluid balance, sedation, nutrition delivery, and rehabilitation readiness. This multidomain approach is consistent with post-critical-illness recovery frameworks (45, 46) and observed functional trajectories in older adults (2). Nutrition guidelines support individualized assessment and early enteral nutrition when appropriate but do not recommend targeting albumin concentrations or composite indices (47, 48). Serial CRP can inform the clinical course but should not replace bedside evaluation (25). Prospective studies should determine whether pattern-informed reassessment changes care or patient-centered outcomes.

Several features strengthen the analysis. The landmark design aligned exposure ascertainment with the start of follow-up. T0 and T1 selection rules and actual sampling times were explicitly reported. The complete operational algorithm was made executable, and 1,000 bootstrap samples re-estimated pooled thresholds, reassigned patterns using fixed directional and count rules, and refitted the models rather than validating only a fixed final model. Frailty and comorbidity were incorporated into the primary analysis, treatment/support variables arising during pattern formation were separated from baseline adjustment, all four patterns were described for secondary outcomes, and negative or model-sensitive findings were reported transparently.

The limitations remain substantial. First, this was a retrospective single-center geriatric ICU study without temporal or external validation. Second, the 72-h landmark excludes early deaths and patients without repeat measurements, so the findings do not apply to all ICU admissions. Third, both pooled thresholds and directional/count rules were internally defined; classification was generally stable in bootstrap analysis, but a small subset of patients near decision boundaries was unstable, and group sizes changed under plausible rule perturbations. Fourth, residual confounding is likely. Albumin, fluid therapy, corticosteroids, antibiotics, nutrition, and organ support can influence the markers; source review confirmed that positive exposures began before T1, but exact patient-level start times, doses, and treatment response were not retained. Fifth, the primary mortality result was sensitive to missing-data and severity-model specifications. Sixth, CFS, Barthel/ADL, NRS-2002, delirium, and T2 measurements were not complete. Seventh, ICU length of stay is affected by the competing processes of death and live discharge, although survivor-restricted and live-discharge analyses were directionally consistent. Finally, correlated component indices cannot identify a specific biological mechanism.

In summary, operational early marker recovery patterns described heterogeneity among critically ill older adults but did not demonstrate an independent association with 28-day mortality after geriatric adjustment or incremental predictive value. Exploratory overall associations with prolonged ventilation and prolonged ICU stay require validation; the ventilation estimates were non-monotonic across patterns. External validation and prospective assessment of pattern-informed reassessment are required.

## 5 Conclusion

Early inflammatory and nutritional marker recovery patterns described clinically relevant heterogeneity during the first 72 h of ICU care. The four-level pattern was not independently associated with 28-day mortality after geriatric adjustment, and it did not improve optimism-corrected prediction. Exploratory overall associations with prolonged ventilation and prolonged ICU stay require external validation. The framework should not be used as a prognostic tool or treatment target without external validation and prospective evaluation.

## Data Availability

The original contributions presented in the study are included in the article/Supplementary material, further inquiries can be directed to the corresponding author.
