# Supplementary material for: Early inflammatory and nutritional marker recovery patterns and short-term outcomes in critically ill older adults: a 72-h landmark cohort study
Source: Front Med (Lausanne). 2026 Sep 4;13:1919790. doi: 10.3389/fmed.2026.1919790 (PMC13587795; doi:10.3389/fmed.2026.1919790)
Supplement: Supplementary file 1 [file Supplementary_File_1.docx]

**Supplementary Materials**

# 1. Supplementary Tables

Supplementary Table S1. Complete operational rules for the four recovery patterns

| **Pattern** | **T0 criterion** | **T1/change criterion** | **Operational interpretation** |
| --- | --- | --- | --- |
| Pattern 1 | T0 extreme-marker count = 0 | Worsening signals <3 and T1 extreme-marker count <2 | No initial extreme burden; delayed-deterioration rule not met |
| Pattern 2 | T0 extreme-marker count >=1 | Improvement signals >=2 and worsening signals = 0 | Initial extreme burden; improvement rule met |
| Pattern 3 | T0 extreme-marker count >=1 | Pattern 2 improvement rule not fulfilled | Initial extreme burden; improvement rule not met |
| Pattern 4 | T0 extreme-marker count = 0 | Worsening signals >=3 or T1 extreme-marker count >=2 | No initial extreme burden; delayed-deterioration rule met |

Note. The categories are investigator-defined operational recovery patterns, not data-derived biological phenotypes. Assignment did not use outcome status.

Supplementary Table S2. Marker thresholds and directional change signals used for pattern construction

| **Marker** | **Extreme-marker criterion** | **Improvement signal** | **Worsening signal** |
| --- | --- | --- | --- |
| CAR | > 4.94 | Relative change <= -25% | Relative change >= +30% |
| NLR | > 19.40 | Relative change <= -20% | Relative change >= +25% |
| SII | > 3281.7 | Relative change <= -20% | Relative change >= +25% |
| PNI | <= 32.40 | Absolute change >= +1.5 | Absolute change <= -1.5 |
| Albumin, g/L | <= 28.60 | Absolute change >= +0.5 g/L | Absolute change <= -0.8 g/L |

Note. Thresholds were pooled T0-T1 internal quartiles. Relative changes used (T1-T0)/T0 x 100%; PNI and albumin signals used absolute changes. These internal rules require external validation.

Supplementary Table S3. Cohort derivation and analysis populations

| **Stage** | **Item** | **n** |
| --- | --- | --- |
| Screened | Assessed for eligibility | 934 |
| Exclusion | Repeat ICU admission | 43 |
| Exclusion | Age <65 years | 62 |
| Exclusion | Postoperative observation only | 54 |
| Exclusion | ICU stay <48 h | 47 |
| Exclusion | Death within 24 h | 21 |
| Exclusion | Death between 24 and 72 h | 9 |
| Exclusion | Hematologic malignancy or severe immunosuppression | 18 |
| Exclusion | Transferred out or outcome unavailable | 12 |
| Exclusion | Missing T0 core CBC and/or albumin | 16 |
| Exclusion | Missing T1 core laboratory measurements | 32 |
| Analysis | Final 72-h landmark cohort | 620 |
| Analysis | 28-day deaths after landmark | 122 |
| Analysis | Primary complete cases | 596 |
| Analysis | Deaths in primary complete-case model | 119 |

Note. Exclusions were applied sequentially. CBC, complete blood count; ICU, intensive care unit; T0, 0-24 h; T1, 48-72 h.

Supplementary Table S4. Laboratory sampling times and within-window selection rules

| **Timepoint** | **Selection rule** | **Available N** | **Median hours** | **IQR low** | **IQR high** | **Minimum hours** | **Maximum hours** |
| --- | --- | --- | --- | --- | --- | --- | --- |
| T0 | 0-24 hours; first measurement in window | 620 | 3.0 | 1.9 | 4.6 | 0.4 | 23.3 |
| T1 | 48-72 hours; first measurement in window | 620 | 58.0 | 54.0 | 62.125 | 48.0 | 72.0 |
| T2 | Day 5-7; exploratory only | 372 | 143.5 | 131.25 | 155.6 | 120.0 | 167.9 |

Note. T0 and T1 used the first available measurement in the window. T2 was exploratory only.

Supplementary Table S5. Missingness summary for key analysis variables

| **Variable/domain** | **Missing n** | **Missing %** |
| --- | --- | --- |
| T0/T1 CAR, PNI, NLR, SII; recovery pattern; primary outcome; age; sex; SOFA; suspected infection; CCI | 0 | 0.0 |
| Body mass index | 38 | 6.1 |
| APACHE II score | 17 | 2.7 |
| Clinical Frailty Scale | 24 | 3.9 |
| Pre-ICU Barthel Index/ADL dependency | 54 | 8.7 |
| NRS-2002 | 77 | 12.4 |
| ICU delirium assessment | 49 | 7.9 |
| T2 CAR, PNI, NLR, and SII | 248 | 40.0 |

Note. T2 measurements were not used for pattern construction or primary modeling. CCI, Charlson Comorbidity Index; SOFA, Sequential Organ Failure Assessment.

Supplementary Table S6. Pre-T1 treatment and organ support by recovery pattern

| **Treatment or support** | **Overall** | **Missing n** | **Pattern 1** | **Pattern 2** | **Pattern 3** | **Pattern 4** | **P value** |
| --- | --- | --- | --- | --- | --- | --- | --- |
| Invasive mechanical ventilation initiated before T1 | 363 (58.5%) | 0 | 131 (51.4%) | 80 (70.2%) | 120 (66.7%) | 32 (45.1%) | <0.001 |
| Vasopressor initiated before T1 | 249 (40.2%) | 0 | 63 (24.7%) | 61 (53.5%) | 105 (58.3%) | 20 (28.2%) | <0.001 |
| CRRT initiated before T1 | 86 (13.9%) | 0 | 21 (8.2%) | 22 (19.3%) | 35 (19.4%) | 8 (11.3%) | 0.002 |
| Albumin infusion initiated before T1 | 157 (25.3%) | 0 | 44 (17.3%) | 43 (37.7%) | 57 (31.7%) | 13 (18.3%) | <0.001 |
| Systemic corticosteroid initiated before T1 | 182 (29.4%) | 0 | 40 (15.7%) | 30 (26.3%) | 91 (50.6%) | 21 (29.6%) | <0.001 |
| Antibiotic therapy initiated before T1 | 494 (79.7%) | 0 | 190 (74.5%) | 98 (86.0%) | 157 (87.2%) | 49 (69.0%) | <0.001 |
| Enteral nutrition initiated within 48 h | 380 (61.3%) | 0 | 160 (62.7%) | 81 (71.1%) | 97 (53.9%) | 42 (59.2%) | 0.028 |
| Fluid balance during first 24 h, mL | 1000.0 [400.0, 1650.0] | 0 | 800.0 [300.0, 1400.0] | 1350.0 [700.0, 1975.0] | 1225.0 [650.0, 1800.0] | 900.0 [425.0, 1525.0] | <0.001 |

Note. Source-record review confirmed that positive indicators began before T1 sampling; exact patient-level start timestamps were not retained. P values are descriptive.

Supplementary Table S7. Descriptive clinical outcomes for all four recovery patterns

| **Outcome** | **Pattern 1** | **Pattern 2** | **Pattern 3** | **Pattern 4** |
| --- | --- | --- | --- | --- |
| 28-day mortality | 25/255 (9.8%) | 26/114 (22.8%) | 57/180 (31.7%) | 14/71 (19.7%) |
| ICU LOS >14 days | 18/255 (7.1%) | 28/114 (24.6%) | 64/180 (35.6%) | 17/71 (23.9%) |
| ICU delirium | 45/237 (19.0%) | 36/106 (34.0%) | 61/163 (37.4%) | 23/65 (35.4%) |
| ICU mortality | 12/255 (4.7%) | 13/114 (11.4%) | 37/180 (20.6%) | 8/71 (11.3%) |
| In-hospital mortality | 21/255 (8.2%) | 20/114 (17.5%) | 53/180 (29.4%) | 14/71 (19.7%) |
| Mechanical ventilation >7 days | 29/131 (22.1%) | 16/80 (20.0%) | 61/120 (50.8%) | 7/32 (21.9%) |

Note. Denominators differ for prolonged ventilation and delirium according to eligibility and assessability.

Supplementary Table S8. Adjusted exploratory secondary outcomes for all four recovery patterns

| **Outcome** | **N/events** | **Pattern** | **OR (95% CI)** | **P value** | **Overall LR P** | **Overall FDR P** |
| --- | --- | --- | --- | --- | --- | --- |
| ICU mortality | 596/68 | Pattern 2 | 0.95 (0.37-2.45) | 0.918 | 0.325 | 0.325 |
|  |  | Pattern 3 | 1.65 (0.74-3.71) | 0.223 |  |  |
|  |  | Pattern 4 | 1.81 (0.64-5.15) | 0.263 |  |  |
| In-hospital mortality | 596/105 | Pattern 2 | 0.89 (0.41-1.92) | 0.770 | 0.102 | 0.128 |
|  |  | Pattern 3 | 1.63 (0.85-3.11) | 0.142 |  |  |
|  |  | Pattern 4 | 2.02 (0.90-4.56) | 0.089 |  |  |
| Mechanical ventilation >7 days | 353/112 | Pattern 2 | 0.49 (0.23-1.07) | 0.072 | <0.001 | 0.002 |
|  |  | Pattern 3 | 1.87 (0.996-3.51) | 0.052 |  |  |
|  |  | Pattern 4 | 0.83 (0.31-2.22) | 0.717 |  |  |
| ICU delirium | 548/159 | Pattern 2 | 1.52 (0.85-2.70) | 0.156 | 0.063 | 0.106 |
|  |  | Pattern 3 | 1.58 (0.94-2.67) | 0.086 |  |  |
|  |  | Pattern 4 | 2.26 (1.21-4.22) | 0.010 |  |  |
| ICU LOS >14 days | 596/123 | Pattern 2 | 2.23 (1.11-4.50) | 0.025 | <0.001 | 0.001 |
|  |  | Pattern 3 | 3.43 (1.83-6.43) | <0.001 |  |  |
|  |  | Pattern 4 | 3.64 (1.70-7.82) | <0.001 |  |  |

Note. Pattern 1 was the reference. Models included all four patterns and adjusted for age, sex, admission year, SOFA, suspected infection at admission, CFS, and CCI. Overall P values are 3-degree-of-freedom likelihood-ratio tests of the three recovery-pattern indicators. Benjamini-Hochberg correction was applied across the five overall tests. Pattern-specific P values are exploratory and were not separately adjusted for multiplicity.

Supplementary Table S9. Sensitivity analyses for 28-day mortality

| **Analysis** | **N** | **Events** | **HR (95% CI)** | **P value** |
| --- | --- | --- | --- | --- |
| Primary geriatric-adjusted model | 596 | 119 | 1.64 (0.97-2.79) | 0.066 |
| Additional pre-T1 organ-support adjustment | 596 | 119 | 1.66 (0.97-2.82) | 0.063 |
| Extended pre-T1 treatment/support adjustment | 596 | 119 | 1.72 (0.99-2.98) | 0.053 |
| Excluding withdrawal or DNR/DNI | 540 | 92 | 1.78 (0.99-3.20) | 0.053 |
| Excluding pre-T1 albumin infusion | 442 | 82 | 1.82 (0.98-3.38) | 0.056 |
| Excluding pre-T1 systemic corticosteroid use | 417 | 73 | 2.07 (1.08-3.98) | 0.029 |
| APACHE II replacing SOFA | 580 | 117 | 2.09 (1.25-3.48) | 0.005 |
| Additional adjustment for sepsis recorded during ICU stay | 596 | 119 | 1.62 (0.94-2.78) | 0.084 |
| T0-only quartile thresholds with fixed directional rules | 596 | 119 | 1.62 (0.96-2.75) | 0.071 |
| Admission year modeled categorically | 596 | 119 | 1.64 (0.96-2.78) | 0.069 |
| Extended ICU-course covariate model | 596 | 119 | 1.63 (0.95-2.79) | 0.078 |
| Multiple imputation (20 datasets) | 620 | 122 | 1.79 (1.06-3.01) | 0.029 |
| T1 burden score, per SD | 596 | 119 | 1.22 (0.93-1.59) | 0.154 |
| Delta PNI, per SD | 596 | 119 | 0.96 (0.76-1.22) | 0.745 |

Note. Unless otherwise stated, estimates compare Pattern 3 with Pattern 1. The primary complete-case model remains the principal analysis; statistically significant sensitivity estimates indicate model dependence rather than confirmatory evidence.

Supplementary Table S10. Directional and count-rule perturbation analyses

| **Scenario and count rule** | **Pattern 1 n** | **Pattern 2 n** | **Pattern 3 n** | **Pattern 4 n** | **HR (95% CI)** | **P** |
| --- | --- | --- | --- | --- | --- | --- |
| Lenient directional thresholds; improvement >= 2; worsening >= 3 | 241 | 109 | 185 | 85 | 1.57 (0.92-2.66) | 0.097 |
| Lenient directional thresholds; improvement >= 2; worsening >= 2 | 192 | 109 | 185 | 134 | 1.59 (0.90-2.81) | 0.111 |
| Lenient directional thresholds; improvement >= 3; worsening >= 3 | 241 | 84 | 210 | 85 | 1.40 (0.83-2.37) | 0.211 |
| Lenient directional thresholds; improvement >= 3; worsening >= 2 | 192 | 84 | 210 | 134 | 1.42 (0.81-2.49) | 0.226 |
| Primary directional thresholds; improvement >= 2; worsening >= 3 | 255 | 114 | 180 | 71 | 1.64 (0.97-2.79) | 0.066 |
| Primary directional thresholds; improvement >= 2; worsening >= 2 | 206 | 114 | 180 | 120 | 1.58 (0.91-2.77) | 0.107 |
| Primary directional thresholds; improvement >= 3; worsening >= 3 | 255 | 78 | 216 | 71 | 1.44 (0.85-2.43) | 0.179 |
| Primary directional thresholds; improvement >= 3; worsening >= 2 | 206 | 78 | 216 | 120 | 1.38 (0.79-2.41) | 0.255 |
| Strict directional thresholds; improvement >= 2; worsening >= 3 | 267 | 100 | 194 | 59 | 1.51 (0.91-2.53) | 0.112 |
| Strict directional thresholds; improvement >= 2; worsening >= 2 | 226 | 100 | 194 | 100 | 1.55 (0.90-2.68) | 0.116 |
| Strict directional thresholds; improvement >= 3; worsening >= 3 | 267 | 62 | 232 | 59 | 1.33 (0.80-2.22) | 0.271 |
| Strict directional thresholds; improvement >= 3; worsening >= 2 | 226 | 62 | 232 | 100 | 1.36 (0.79-2.34) | 0.266 |

Note. Each scenario reclassified patients and refitted the primary geriatric-adjusted Cox model. HRs compare Pattern 3 with Pattern 1. These analyses quantify investigator-rule sensitivity.

Supplementary Table S11. Internal bootstrap stability after threshold re-estimation and reclassification

| **Marker** | **Original** | **Median** | **2.5%** | **97.5%** |
| --- | --- | --- | --- | --- |
| CAR | 4.94 | 4.93 | 4.37 | 5.47 |
| NLR | 19.40 | 19.40 | 17.52 | 22.00 |
| SII | 3281.7 | 3281.3 | 2996.3 | 3742.4 |
| PNI | 32.4 | 32.4 | 31.8 | 32.9 |
| Albumin | 28.6 | 28.6 | 28.3 | 29.0 |

Overall agreement: median 0.984 (95% interval, 0.958-0.995); Cohen kappa 0.977 (0.941-0.993). Patient-level stability <0.80 occurred in 24/620 patients; minimum stability was 0.279. In each bootstrap sample, pooled thresholds were re-estimated, patterns were reassigned using the fixed directional and count rules, and the Cox and prediction models were refitted.

Supplementary Table S12. Proportional-hazards diagnostics for the primary Cox model

| **Term** | **df** | **Chi-square** | **P value** | **Diagnostic scope** | **Time transform** |
| --- | --- | --- | --- | --- | --- |
| Pattern 2 | 1 | 0.007 | 0.932 | Individual coefficient | Kaplan-Meier |
| Pattern 3 | 1 | 0.033 | 0.855 | Individual coefficient | Kaplan-Meier |
| Pattern 4 | 1 | 1.726 | 0.189 | Individual coefficient | Kaplan-Meier |
| Age | 1 | 0.097 | 0.755 | Individual coefficient | Kaplan-Meier |
| Male sex | 1 | 2.109 | 0.146 | Individual coefficient | Kaplan-Meier |
| Admission year | 1 | 0.942 | 0.332 | Individual coefficient | Kaplan-Meier |
| SOFA score within 24 h | 1 | 2.488 | 0.115 | Individual coefficient | Kaplan-Meier |
| Suspected infection at admission | 1 | 1.340 | 0.247 | Individual coefficient | Kaplan-Meier |
| Clinical Frailty Scale | 1 | 0.060 | 0.806 | Individual coefficient | Kaplan-Meier |
| Charlson Comorbidity Index | 1 | 0.018 | 0.894 | Individual coefficient | Kaplan-Meier |
| Recovery pattern (joint) | 3 | 2.536 | 0.469 | Joint term | Kaplan-Meier |
| Global diagnostic | 10 | 8.832 | 0.548 | Global | Kaplan-Meier |

Note. Neither the recovery-pattern block nor the global diagnostic indicated violation of the proportional-hazards assumption.

Supplementary Table S13. Exploratory prediction performance and incremental tests

| **Model** | **N** | **Events** | **Apparent AUROC (95% CI)** | **Corrected AUROC** | **Corrected Brier** | **Corrected intercept** | **Corrected slope** |
| --- | --- | --- | --- | --- | --- | --- | --- |
| Clinical | 596 | 119 | 0.730 (0.679-0.781) | 0.717 | 0.145 | 0.008 | 0.938 |
| Clinical + geriatric | 596 | 119 | 0.761 (0.710-0.812) | 0.747 | 0.134 | 0.008 | 0.932 |
| Clinical + geriatric + recovery pattern | 596 | 119 | 0.767 (0.716-0.818) | 0.745 | 0.134 | 0.009 | 0.900 |

Incremental tests: Clinical vs clinical + geriatric - Paired DeLong: P=0.091; Clinical + geriatric vs clinical + geriatric + recovery pattern - Paired DeLong: P=0.365; Clinical vs clinical + geriatric - Likelihood-ratio: P<0.001; Clinical + geriatric vs clinical + geriatric + recovery pattern - Likelihood-ratio: P=0.231. Corrected metrics used 1,000 bootstrap samples. Prediction analyses were exploratory and internally validated only.

Supplementary Table S14. Robustness analyses for ICU length of stay

| **Analysis** | **N** | **Events** | **Effect measure** | **Effect (95% CI)** | **P value** |
| --- | --- | --- | --- | --- | --- |
| ICU LOS >14 days among ICU survivors | 371 | 62 | OR | 3.91 (1.89-8.09) | <0.001 |
| ICU LOS >14 days among day-28 survivors | 338 | 57 | OR | 4.49 (2.09-9.65) | <0.001 |
| Continuous ICU LOS among ICU survivors | 371 | — | Geometric mean ratio | 1.34 (1.20-1.50) | <0.001 |
| Cause-specific hazard of live ICU discharge (ICU death censored) | 419 | 371 | Cause-specific HR | 0.52 (0.41-0.67) | <0.001 |

Note. Analyses compare Pattern 3 with Pattern 1. ICU death and live discharge are competing processes; the cause-specific model censors ICU death.

# 2. Supplementary Figures

**Supplementary Figure S1. Empirical cumulative distributions of core inflammatory and nutritional markers at T0 and T1 by recovery pattern. CAR, NLR, and SII are shown after log10 transformation; PNI is shown on the original scale.**

**Supplementary Figure S2. Spearman correlation structure of T0 and T1 CAR, NLR, SII, and PNI with SOFA score, Clinical Frailty Scale, and Charlson Comorbidity Index.**

**Supplementary Figure S3. Exploratory prediction assessment. Panel A shows receiver-operating-characteristic curves. Panel B shows apparent calibration by risk decile. Panel C shows decision-curve analysis. Models were not externally validated.**

**Supplementary Figure S4. Internal bootstrap stability after threshold re-estimation and reclassification. Panel A shows threshold distributions; Panel B patient-level reference-pattern stability; Panel C whole-cohort agreement and Cohen kappa; Panel D Cox estimate distributions. Fixed directional and count rules were used within each bootstrap sample. This is internal stability, not external reproducibility.**

**Supplementary Figure S5. Exploratory T2 marker trajectories among patients with available day 5-7 measurements. T2 was not used for recovery-pattern construction or primary modeling, and selective availability limits interpretation.**
